# Astaxanthin Inhibits PC-3 Xenograft Prostate Tumor Growth in Nude Mice

**DOI:** 10.3390/md15030066

**Published:** 2017-03-08

**Authors:** Xiaofeng Ni, Haining Yu, Shanshan Wang, Chengcheng Zhang, Shengrong Shen

**Affiliations:** 1 Department of Food Science and Nutrition, College of Biosystems Engineering and Food Science, Zhejiang University, Hangzhou 310058, China; contrabutt@126.com (X.N.); 11413031@zju.edu.cn (S.W.); zccwsf@126.com (C.Z.); 2 College of Pharmaceutical Science, Zhejiang University of Technology, Hangzhou 310014, China; yuhaining@zjut.edu.cn

**Keywords:** prostate cancer, astaxanthin, immunohistochemistry, PCR-DGGE, miRNA

## Abstract

Prostate cancer (PCa), the most common malignancy in men, is a major cause of cancer deaths. A better understanding of the mechanisms that drive tumor initiation and progression may identify actionable targets to improve treatment of this patient group. As a dietary carotenoid, astaxanthin has been demonstrated to exert beneficial effects against inflammation, cardiovascular disease, oxidative damage, or different cancer sites. This study used intragastric administration of astaxanthin to detect its role on tumor proliferation, apoptosis, microRNA (miRNA) overexpression, and microbacteria composition change by establishing androgen-independent PCa cell PC-3 xenograft nude mice. Nude mice were inoculated with androgen-independent prostate cancer PC-3 cells subcutaneously. The intervention was started when tumors reached 0.5–0.6 cm in diameter. Mice were intragastrically administered 100 mg/kg astaxanthin (HA), 25 mg/kg astaxanthin (LA), or olive oil (TC). The results showed that 100 mg/kg astaxanthin significantly inhibited tumor growth compared to the TC group, with an inhibitory rate of 41.7%. A decrease of Ki67 and proliferating cell nuclear antigen (PCNA) as well as an increase of cleaved caspase-3 were observed in HA-treated tumors, along with increasing apoptotic cells, obtained by TUNEL assay. The HA significantly elevated the levels of tumor suppressors miR-375 and miR-487b in tumor tissues and the amount of *Lactobacillus* sp. and *Lachnospiraceae* in mice stools, while there was no significant difference between LA and TC groups. These results provide a promising regimen to enhance the therapeutic effect in a dietary supplement manner.

## 1. Introduction

Prostate cancer (PCa), the most common malignancy in men, is one of the leading causes of cancer deaths in the world. As a result, in 2015, about 27,500 men died of this disease and an estimated 220,800 men were diagnosed with PCa in the United States [1]. From the clinical point of view, the overall 5-year survival rate is less than 5% [2]. Because of its invasive and metastatic characteristics, less than 10% of patients are eligible for surgery at the time of diagnosis because the poor prognosis of the disease is related to late presentation, aggressive local invasion, and early metastasis. Conventional chemotherapy and radiotherapy are generally ineffective, and the emergence of drug resistance is also common [3,4].

The 5-alpha-reductase inhibitors, finasteride and dutasteride, which have a potent ability to block the conversion of testosterone to dihydrotestosterone, have been studied for PCa chemoprevention in large, phase III chemoprevention trials [5,6,7]. Although these agents significantly reduced the risk of PCa, these agents are still not widely used for PCa chemoprevention because their use was also associated with increased detection of high-grade disease.

Many natural compounds have been investigated for their ability to slow down or possibly stop the natural course of PCa. The most widely studied compounds have been vitamin E, selenium, lycopenes, polyphenols, and isoflavones. All these compounds yield multiple in vitro and in vivo anticarcinogenic features, such as antioxidation, induction of apoptosis, and inhibition of cellular proliferation and angiogenesis [8]. These properties, combined with their low toxicity, make them ideal candidates for PCa chemoprevention.

The potential cancer protective effect of diets rich in antioxidants has been extensively studied in relation to different cancer sites [9,10,11]. Amongst those studies, astaxanthin (3,3′-dihydroxy-β-β′-carotene-4,4′-dione) is one of the compounds attracting growing interest. Astaxanthin, a carotenoid with antioxidant properties, is 100–1000 times more effective than the well-known vitamin E. Dietary supplementation with astaxanthin was reported to exert beneficial effects against inflammation, cardiovascular disease, and oxidative damage, suggesting that astaxanthin is a functional food ingredient [12,13,14]. The European Commission considers natural astaxanthin as a food dye [15]. The consumption of astaxanthin can prevent or reduce risk of various disorders in humans and animals [16,17], however, there is no study focusing on the mechanism of astaxanthin’s effects on animal xenografts of PCa.

The development of PCa is a long-term process driven by genetic and epigenetic changes and characterized by abnormal cell and tissue differentiation. Hence, a better understanding of the mechanisms that drive tumor initiation and progression may identify actionable targets to improve treatment of this patient group.

This research used intragastric administration of astaxanthin to detect its role on tumor proliferation, apoptosis, microRNA (miRNA) overexpression, and microbacteria composition change by establishing androgen-independent PCa cell PC-3 xenograft nude mice.

## 2. Results

### 2.1. Suppressed Tumor Growth of PC-3 Xenograft

**Figure 1:**
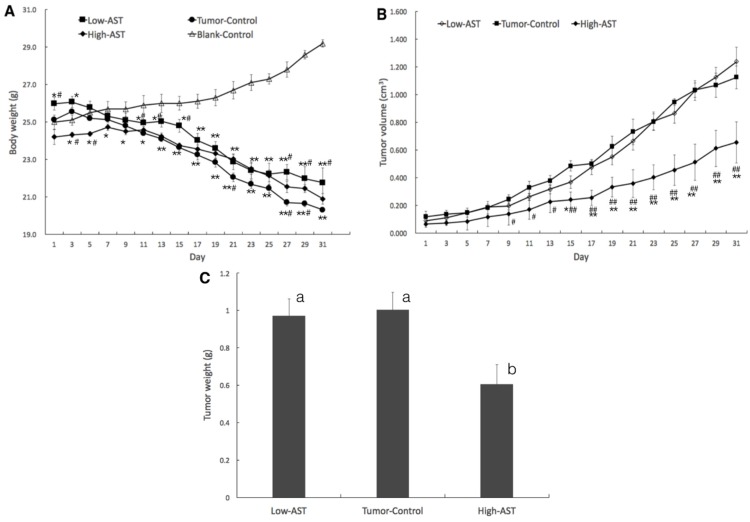
High-dose astaxanthin inhibits the growth of PC-3 tumor xenografts in nude mice. PC-3 cells (1 × 10^6^ cells) were subcutaneously implanted into the anterior axillary of nude mice. Tumor-bearing mice were treated with astaxanthin (25 or 100 mg/kg·day) for 31 days, when tumor volume reached 5–6 mm in diameter. Body weight (**A**); * *p* < 0.05, ** *p* < 0.01 compared to the blank control, # *p* < 0.05, ## *p* < 0.01 compared to the tumor control; tumor volume (**B**); # *p* < 0.05 compared to the tumor control, * *p* < 0.05, ** *p* < 0.01 compared to the low-dose astaxanthin; and tumor weight (**C**) were recorded every 2 days. Values with different letters are significantly different (*p* < 0.05).

After 1 week, a small bump could be felt under the armpit but with no significant difference among the groups. All of the nude mice developed tumors after 2 weeks with diameters of 5–6 mm, and we began to record the body weight and tumor volume. After 15 days, body weight gradually decreased in tumor groups, though no significant difference was noted, while body weight slowly increased in blank control (equivalent volume of olive oil, BC) group (Figure 1A). From day 17, the tumor volume of the high-astaxanthin (100 mg/kg·day astaxanthin, HA) group was significant smaller than the low-astaxanthin (25 mg/kg·day astaxanthin, LA) and tumor control (equivalent volume of olive oil, TC) group (*p* < 0.01). At the 31st day of the interventional experiment, the average tumor volume was 1126 mm^3^ in the TC group, but 656 mm^3^ in the HA group, indicating 41.7% inhibition of tumor regrowth with 100 mg/kg·day astaxanthin (Figure 1B). The tumor weight in the HA group was significantly smaller than that in the TC and LA groups (*p* < 0.05), while the inhibitory rate was 39.6% (Figure 1C). However, there was no significant difference of tumor volume and weight between LA and TC group.

### 2.2. Decreased Tumor Cell Proliferation and Increased Tumor Cell Apoptosis in Tumors

**Figure 2:**
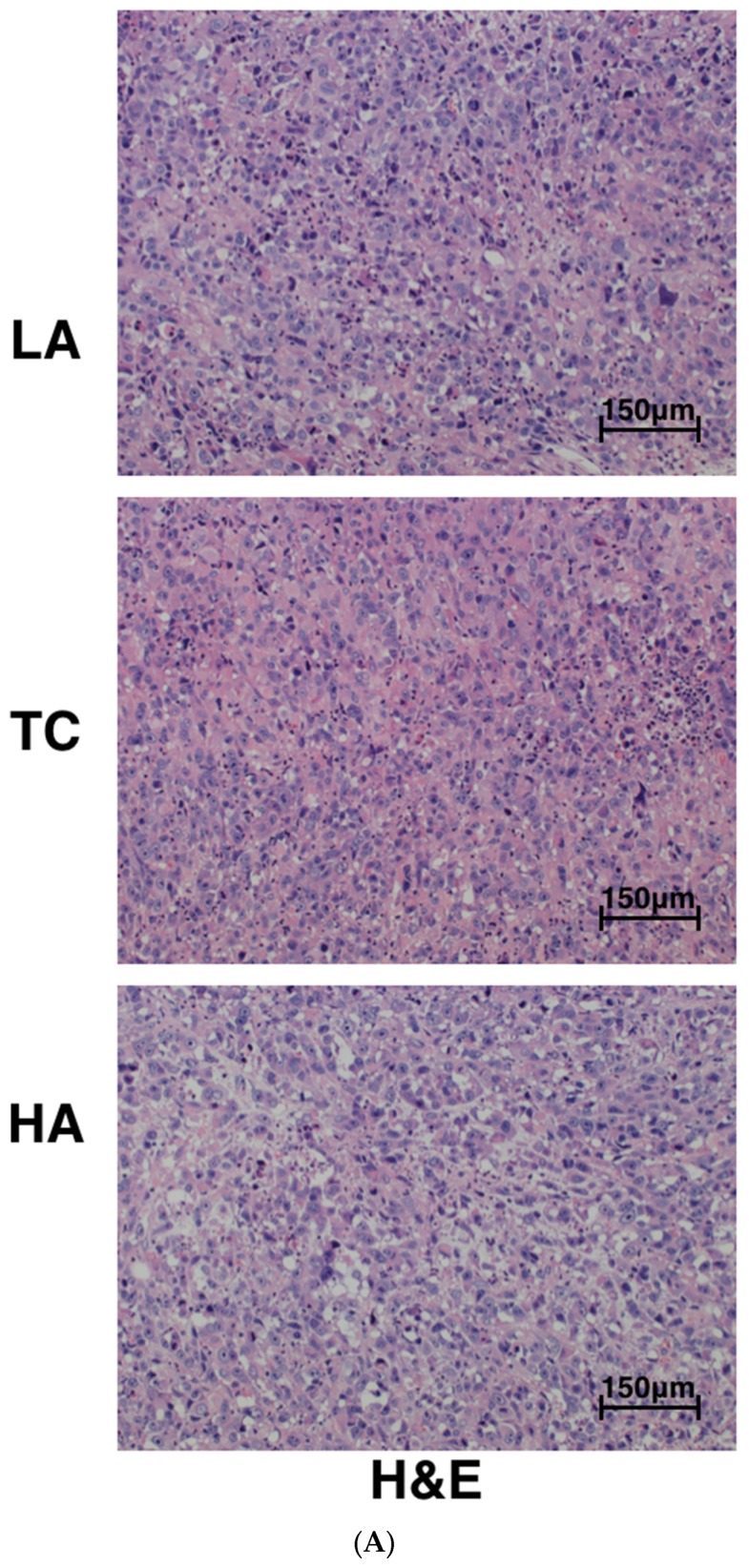

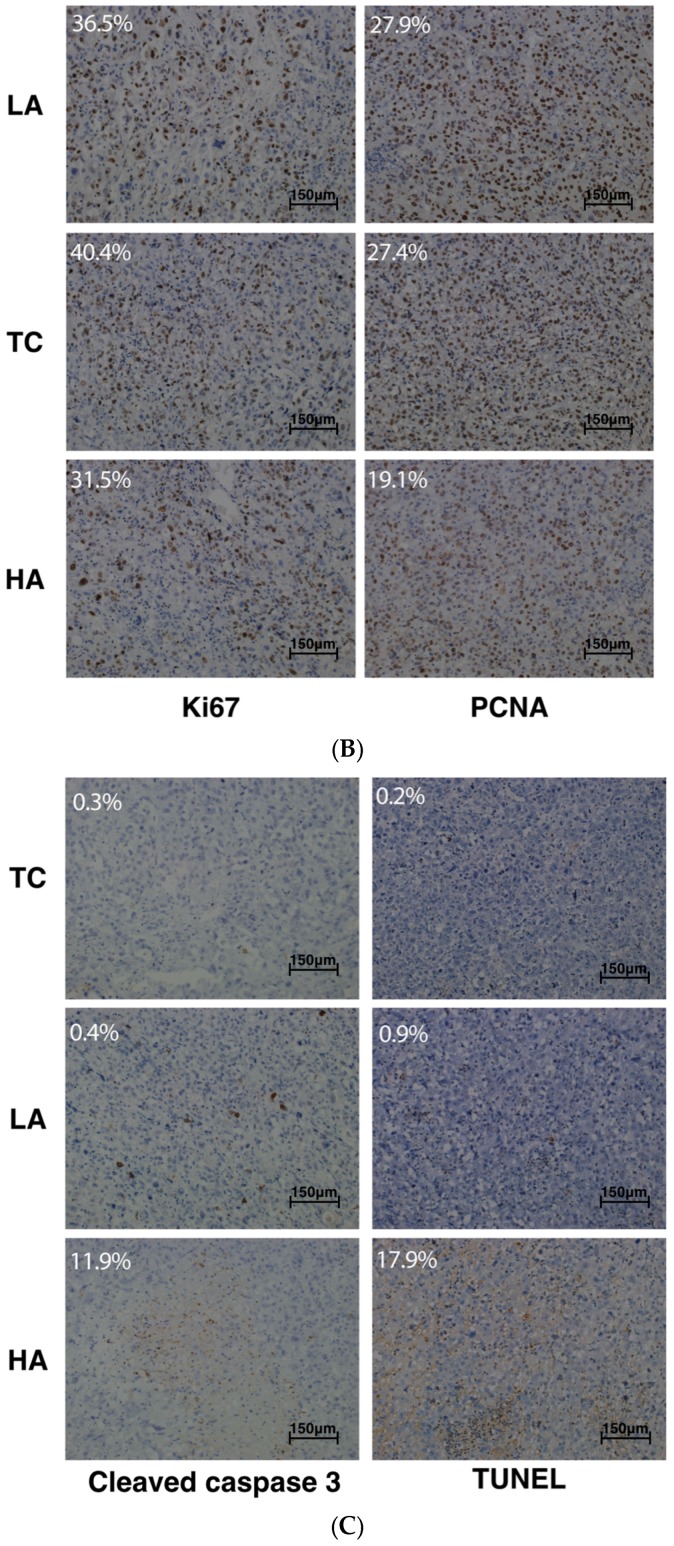
Immunohistochemical analysis of PC-3 xenografts in nude mice treated with astaxanthin. Tumors from control and astaxanthin-treated mice were sectioned and stained with hematoxylin and eosin (H&E) (**A**); to examine the effects of astaxanthin on cell proliferation by using anti-PCNA (proliferating cell nuclear antigen), anti-Ki67 (**B**); and cell apoptosis by using anti-cleaved caspase-3, TUNEL assay (**C**) in tumors. Percentage represents positive ratio. LA: low-astaxanthin (25 mg/kg·day); TC: tumor control; HA: high-astaxanthin (100 mg/kg·day).

Tumors from controls and astaxanthin-treated animals were sectioned and stained with hematoxylin and eosin (H&E) (Figure 2A). Histopathological analysis revealed that tumors from control-treated mice were uniformly composed of living cells with a small population of dead cells. In contrast, tumors from HA-treated mice had large amounts of tumor cell necrosis.

We further examined the effects of astaxanthin on cell proliferation and apoptosis in tumor tissues derived from control and astaxanthin-treated mice using anti-PCNA (proliferating cell nuclear antigen), anti-Ki67, cleaved caspase-3 antibody, and the TUNEL assay, respectively. PCNA and Ki67 are the markers of cell proliferation, while cleaved caspase-3 and fragmented DNA (detected by the TUNEL assay) are markers of cell apoptosis.

As shown in Figure 2B, these cell proliferation indexes were dramatically reduced in HA-treated mice tumors compared to the TC and LA groups. In addition, the HA group caused an induction of cleaved caspase-3 and apoptotic cells in the tumors, respectively (Figure 2C). However, no significant differences were identified between LA and TC group.

These data are consistent with the tumor growth curve as measured by tumor volume (Figure 1B), indicating that inhibition of cell proliferation and induction of apoptosis may underlie HA-induced inhibition of PCa relapse after androgen deprivation therapy.

### 2.3. Modulation of Bacterial Composition in Mice Stools

**Figure 3:**
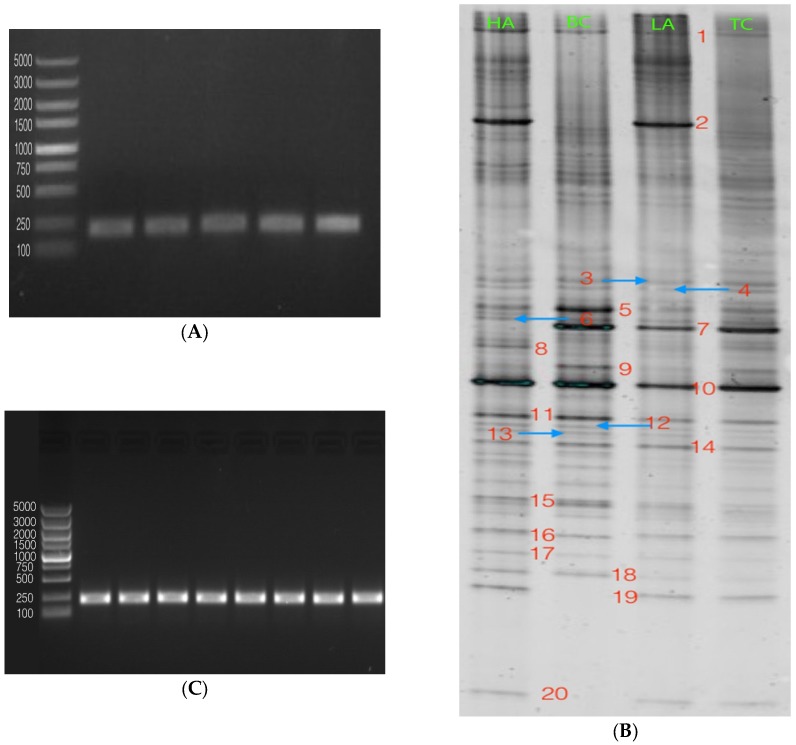

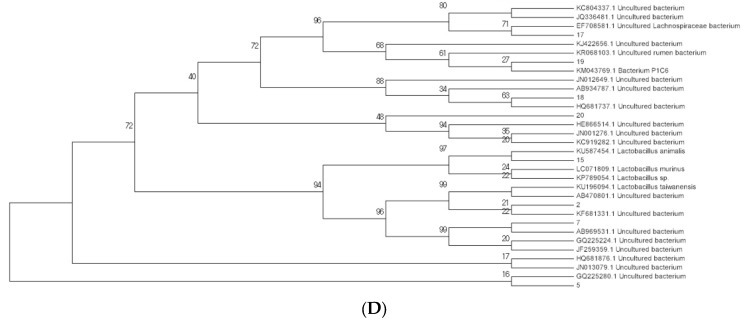
PCR-DGGE (denaturing gradient gel electrophoresis) analysis of stools in nude mice treated with astaxanthin. The total genomic DNA was isolated from the mice stools and amplified to 250 bp fragments (**A**); the DNA fragments with different sequences were separated by DGGE electrophoresis, graphs were digitized by Quantity One Analysia software (**B**); selected DGGE bands were cut, purified, amplified (**C**) and sequenced, and obtained sequences were BLASTed to find the closest relative for the partial 16S rRNA gene. Based on the BLAST results, reference sequences of phylogenetic neighbor species (up to 98% similarity) were included for constructing phylogenetic tree (**D**).

The total DNA of each group was successfully extracted, and the amplified fragments of PCR were approximately 250 bp, as shown in Figure 3A. Based on the results obtained, it is evident that 16S rRNA gene fragments from bacteria in the stools had good amplification efficiency.

The results of denaturing gradient gel electrophoresis (DGGE) fingerprinting with primer pair F341-GC and R518, which amplified the total microbial community, are presented with separated bands of different DNA sequences in Figure 3B; about 20 discernible bands were observed. According to the DGGE profile, bands 2, 5, 7, 15 and 17–20 were selected for sequencing because there was obvious difference among different groups. The eight bands were reamplified (Figure 3C) and the resulting sequences of these bands matched with *Lactobacillus* sp., uncultured Deferribacterales bacteria, *Lactobacillus*, *Lactobacillus* sp., *Lachnospiraceae*, uncultured bacteria, *Lachnospiraceae*, and uncultured Bacteroidales bacteria, respectively. The evidence from these results could lead to the conclusion that there were two main species of *Lactobacillus* and *Lachnospiraceae* in the diversity of bacteria among the groups. *Lactobacillus* sp. was present in higher amounts in astaxanthin-treated groups, specifically in the HA group. In addition, *Lachnospiraceae* was also present in higher amounts in the HA group compared with other tumor groups. Uncultured Deferribacterales bacteria was only found in the nontumor control group.

Sequences of the bands were then BLASTed in the NCBI database; a sequence having above 98% similarity was regarded as the same. Among these strains, the one that showed the highest similarity of the same category was selected. Based on these results, the phylogenetic tree was created by MEGA 7.0.

As shown in the phylogenetic tree (Figure 3D), most of the strains were uncultured, and values on the branches of the tree show a high confidence coefficient and close genetic relationships.

### 2.4. Modulation of miRNA Expression

We examined the expression of 84 miRNAs with microarray analysis in the HA and TC groups. Generally, in PCa, miRNAs are significantly lower in cancer tissues than in adjacent normal tissue [18,19]. Two miRNAs showing overexpression with a fold variation higher than 1.5 were picked out, one had a 1.9-fold increase (miR-375) and the other had a 2.1-fold increase (miR-487b).

**Figure 4:**
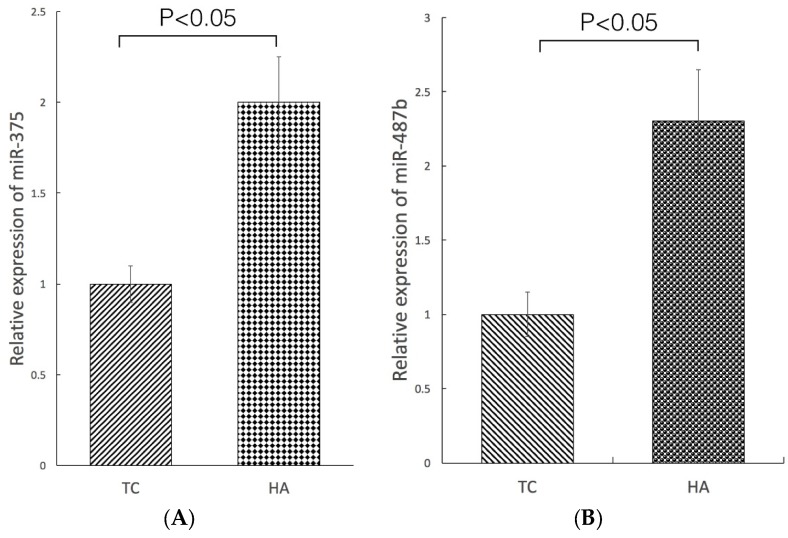
The expression levels of miR-375 and miR-487b in PC-3 xenograft nude mice. Relative expression of miR-375 (**A**) and miR-487b (**B**) was determined by qRT-PCR and corrected to RUN44 levels. Results are representatives of three independent experiments. Data are shown as mean ± SD.

To validate the reliability of the microarray data, qRT-PCR was used to assess the expression levels of both miRNAs (Figure 4A,B). Both miR-375 and miR-487b were significantly overexpressed in the HA group (*p* < 0.05), confirming the results of the array.

## 3. Discussion

As a dietary carotenoid, astaxanthin is extracted from algae, shrimp, salmon, lobster, and some other organisms [20,21]. A large number of studies have shown astaxanthin has strong biological activities including anti-lipid peroxidation, anti-inflammation, immune modulation, and antioxidation [22,23,24]. Emerging data indicate that astaxanthin may play a role in therapy for cancers such as breast cancer, PCa, colon cancer, and neuroblastoma [25,26,27,28]. Nakagawa et al. [29] reported that 12-week astaxanthin supplementation (6 or 12 mg/day) improved erythrocyte antioxidant status and decreased phospholipid hydroperoxide levels in the erythrocytes of 30 middle-aged and senior subjects. Choi et al. [30] reported that supplemental astaxanthin (5 or 20 mg/day) for 3 weeks improved oxidative stress biomarkers (superoxide dismutase and antioxidant capacity) by suppressing lipid peroxidation (malondialdehyde and isoprostane) and stimulating the activity of the antioxidant defense system. However, few studies have been done on the effects of astaxanthin in PC-3 xenograft nude mice.

In this study, significant tumor volume reduction was obtained in the HA group of xenograft nude mice. PC-3 tumor growth was largely inhibited from day 15 in the HA group, while tumor growth was not inhibited in LC group. Among the tumor groups, body weight was significantly decreased. Tumor weight and the rate of body weight loss was minimal in the HA group. These findings signified that the effects were greater at the high dose than at the low dose. Naturally, it is difficult to determine the optimal dose in this present study; it needs further study to figure out an accurate determination of the optimal dose.

Uncontrolled proliferation in the prostate increases the number of stromal and epithelial cells, resulting in enlarged prostate volume. Aberrant cell proliferation is associated with deregulation of the cell cycle, and the expression of cell cycle-regulation proteins has been evaluated to understand how the proliferation is related to various diseases and cancers. The expression of PCNA and Ki67 was increased significantly in xenografts compared with normal nude mice. High-dose astaxanthin-treated mice had significantly reduced PCNA and Ki67 protein levels. Decreased prostate tumor growth was associated with an increase in PCa cell apoptosis, as confirmed by TUNEL staining assay. Our results also indicated that cleaved caspase-3 levels were overexpressed in the HA group compared to the TC group. These findings suggest that the effects of high astaxanthin on tumor development involve antiproliferative activity.

Comprehending the composition and richness of microbiota, which are related to health, is essential for understanding a cause of prostate disease, as well as for the prevention, diagnosis, and treatment. Molecular-based methods to identify and characterize microorganisms have contributed to microbial discovery; 16S rDNA-based PCR assays are more sensitive than the traditional methods that rely on microbial culture techniques [31,32]. Several studies have evaluated the presence of multiple and diverse bacterial 16S rDNA sequences in prostate biopsy tissue from benign prostatic hyperplasia (BPH) and PCa patients [33,34,35,36]. Yu et al. [37] collected expressed prostate secretions (EPSs) to investigate the presence and possible shift in the type and quantity of microbiota in BPH and PCa, as they thought EPSs are secreted by the prostate and pass through the urethra, which is likely how bacteria in the male reproductive tract and urethra could access EPSs.

In this study, we utilized a combination of PCR-DGGE with broad-range primers and sequencing, combined with phylogenetic analysis to investigate microbiota in normal and xenograft nude mice. The sequencing results showed that HA-treated tumor was rich in *Lachnospiraceae* and *Lactobacillus* sp. Previous studies showed that the microbial population in EPS, urine, seminal fluid, and prostate biopsy in PCa patients were significantly different, indicating correlation between PCa and BPH with microbiota [38]. Several studies had reported that lactobacilli enhance natural killer cell (NK) activity to effectively attack malignant tumor cells in vitro and in vivo [39,40,41]. However, there have been few studies on the effect of *Lactobacillus* on prostate cancer. Intestinal flora is related to human health and immunity, but whether there is any connection between urinary tract infection and intestinal flora—since urinary tract infection will change bacterial flora, which we had reported before [37,38]—had not been investigated previously. Whether *Lactobacillus* sp. is involved in the progression of PCa tumor suppression needs more researches to support a conclusion. On the other hand, intestinal flora can be a target for cancer prevention and treatment. Danino et al. developed a method for the prevention of liver cancer metastasis from urine by oral administration through the use of safe and widely used probiotic *Escherichia coli* Nissle 1917 [42]. Further study of the mechanism was necessary.

MiRNA is a small noncoding RNA that post-transcriptionally regulates gene expression through the interaction with targeted mRNAs, leading to the translational repression or cleaving of RNA transcripts in a sequence-specific manner. Recent bioinformatics analyses have shown that miRNA regulates the expression of approximately 60% of all genes. Thus, miRNA is recognized as a mechanism of fine-tuning regulation, and is likely to be involved in almost all biological processes. In cancer, miRNAs are globally downregulated, although some are notoriously upregulated [43]. A growing body of literature in particular has investigated the potential use of miRNAs as useful biomarkers for cancer diagnosis, prognosis, and therapy—including PCa—and associated their levels with clinicopathologic parameters [44,45]. The use of array technologies to characterize miRNAs has enabled discovering unique miRNA expression patterns related to clinical features of several cancers. Thus, assessing miRNAs differential expression in specific patients carrying primary tumors and searching for miRNA targets is critical to investigate their relevance in PCa initiation and progression.

Several studies have shown that the up- and downregulation of miRNA, as determined by analysis of miRNA expression signatures, are associated with PCa pathogenesis [46,47]. Therefore, identification of biomarkers more sensitive than prostate-specific antigen (PSA) is necessary in order to improve outcomes in patients with PCa. In the present study, we found a higher expression of miR-375 and miR-487b in the HA group, which indicated that high concentration of astaxanthin could suppress tumor growth by inducing overexpression of miR-375. Also, several studies have shown that the expression of miR-375 is significantly reduced in tumor tissues, and this miRNA functions as a tumor suppressor by targeting several oncogenic genes [48,49,50,51,52]. On the other hand, miR-487b has an important role as a tumor-suppressive by inhibiting cell proliferation, invasion, and migration, and has been mapped to 14q32.31 in metastatic PCa cells [53]. It is clear that individual miRNAs might have opposing roles in different cell types by targeting different pathways/genes. Therefore, it is necessary to investigate the mechanisms through which miR-375 and miR-487b regulate cancer pathways/genes in different types of cancer cells in the future.

## 4. Materials and Methods

### 4.1. Ethics Statement

The study was approved by Laboratory Animal Management Committee of Zhejiang University and was arranged to minimize suffering and to reduce the number of animals used. All the animals received humane care in compliance with Guide for the Care and Use of Laboratory Animals. All mice were anesthetized with isoflurane and sacrificed for the collection of tumor tissue samples.

### 4.2. Cell Culture

Human PCa cell line PC-3 was bought from the Chinese academy of sciences, Shanghai Institute of Biochemistry and Cell Biology (Shanghai, China). The cells were cultured in RPMI 1640 supplemented with 10% fetal bovine serum and 100 U/mL penicillin–streptomycin. The cells were maintained at 37 °C and 5% CO_2_ in a humid environment.

### 4.3. Animal Model

Four-week-old nude mice were purchased from Slac Laboratory Animal company (Shanghai, China). Mice were housed individually at 24 ± 1 °C in a light-controlled room (light: 7:00–19:00 h, dark: 19:00–7:00 h) and a relative humidity of 55% ± 5% (Laboratory Animal Center, Zhejiang University). Mice were given an ad libitum supply of filtered pathogen-free air, food, and water.

PC-3 cells in logarithmic phase were digested into a single cell suspension at 1 × 10^7^/mL. One hundred microliters of the cell suspension was injected into the left subcutaneous anterior axillary of the 5-week-old nude mice under aseptic condition. Astaxanthin was purchased from Sigma-Aldrich (St. Louis, MO, USA) and the solvent was olive oil. When tumor volume reached 5–6 mm in diameter, 40 nude mice were randomly divided into four groups: BC, TC, HA, and LA. Continuous measurements of tumor dimensions were conducted using a caliper. The tumor diameter (a) and short diameter (b) were measured every 2 days to draw the tumor growth curve. Tumor volume was calculated by the following formula: Tumor volume = a × b^2^/2. On the 31st day, mice were sacrificed, tumors were stripped, and weighed.

### 4.4. Hematoxylin–Eosin (H&E) Staining and Immunohistochemical (IHC) Analyses

A section of each tumor was fixed in 10% formalin for IHC determinations. Sections of 3–4 μm were cut from each sample by deparaffinizing and dehydrating using Hemo-De (Fisher Scientific, Pittsburgh, PA, USA), followed by an alcohol series and washing in phosphate-buffered saline (PBS), pH 7.0. The slides were then incubated in 3% H_2_O_2_ for 20 min at room temperature to block peroxidase, and antigens were retrieved by boiling the slides in a microwave oven in 50 mM citrate buffer, pH 6.4. After blocking with goat serum, the slides were incubated with PCNA, Ki67, and cleaved caspase-3 antibodies (Google Biological Science and Technology Co., Ltd., Wuhan, China). The slides were counterstained with hematoxylin. H&E stained sections were also made. Apoptosis was detected by the TUNEL assay using the DeadEnd^TM^ Colorimetric TUNEL System (Promega, Madison, WI, USA) per manufacturer’s protocol. Three random microscopic fields were captured for each tumor section at 200× magnification.

### 4.5. Polymerase Chain Reaction–Denaturing Gradient Gel Electrophoresis (PCR-DGGE)

The total genomic DNA was isolated from the mice stools according to the instructions of the ZR Fecal DNA MiniPrep^TM^ Kit (Zymo Research, Irvine, CA, USA). The extracted DNA was packed into three tubes to avoid multi-gelation and stored at −20 °C. Each DNA sample used in this study was first amplified with universal bacterial primers. The forward primer 341 (5′-GTATTACCGCGGCTGCTGG-3′) containing a 40 bp GC clamp (5′-CGCCCGCCGCGCGCGGCGGGCGGGGCGGGGGCACGGGGGG-3′) and the reverse primer 518 (5′-ATTACCGCGGCTGCTGG-3′) resulted in fragments of approximately 250 bp to test the quality of the template and to rule out the presence of PCR inhibitors. The GC clamp could increase the sensitivity of the DGGE analysis [54].

DNA fragments with different sequences were separated in 8% polyacrylamide. A denaturing gradient of 40%–60% was applied in the DGGE electrophoresis, formed with deionized formamide and urea. Gels were electrophorized in 1 × TAE buffer under 60 °C and 200 V for 3.5 h. DGGE graphs were digitized by Quantity One Analysia software (Gene Genius, Cambridge, UK).

Selected DGGE bands (2, 5, 7, 15, and 17–20) were cut with a sterile surgical blade under UV light and purified with DNA Gel Extraction Kit (SK1135, Sangon, Shanghai, China). The purified DNA was then amplified again with the primers described previously. The PCR products were ligated into the pUCm–T vector (Sangon, Shanghai, China) and transformed into competent *E. coli* DH5α cells (Sangon). Bacteria were collected and DNA with the plasmid was extracted by UNIQ-10 Column Kit (Sangon) following the kit’s protocol. Clones that migrated to the same position as the original DGGE bands were sequenced (Sangon).

Obtained sequences were searched online based on NCBI GenBank database (http://www.ncbi.nlm.nih.gov) BLAST to find the closest relative for the partial 16S rRNA gene. Based on the BLAST results, reference sequences of phylogenetic neighbor species (up to 98% similarity) were included for constructing phylogenetic tree using the MEGA 6 software package, v6.0, according to the method of neighbor-joining based on evolutionary distances. The consistency of the tree was validated by bootstrapping (*n* = 1000).

### 4.6. Microarray Array

Total RNA was isolated from mice tumor tissue using TRI Reagent (guanidium/phenol, Sigma Bioscience, Shanghai, China). Briefly, tissue samples were homogenized in 1 mL of TRI Reagent, and 0.2 mL of chloroform was added to each sample. The mixtures were shaken for 15 s, followed by centrifugation at 4 °C and 12,000× *g* for 15 min. The organic layer was transferred to a clean 1.5 mL nuclease-free EP tube, and a volume of isopropanol equal to that of the aqueous phase was added to each sample. Then, the aqueous solution was homogenized by vortexing or flicking before resting for more than 30 min at −20 °C, after which the samples were centrifuged at 4 °C and 12,000× *g* for 15–30 min. The RNA pellets were washed with 75% ethanol. RNA was dissolved in RNase-free water. The prepared RNA was checked by electrophoresis to show that the RNA integrity was uncompromised, and then concentration was measured using a NanoDrop ND-2000c (Thermo Fisher Scientific, Inc., Wilmington, DE, USA) by measuring the optical density. The optical density of all RNA samples was 1.8–2.0 based on the 260/280 ratio. RNA was stored at −80 °C.

**Figure 5:**
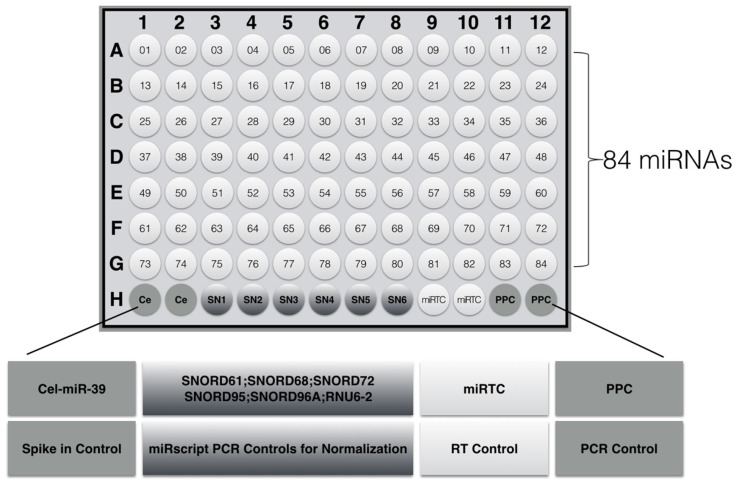
The layout design of miRNA PCR array in 96-well plate. (1) Cel-mir-39: alternative data normalization using exogenously spiked Syn-cel-miR-39 miScript miRNA Mimic; (2) miScript PCR Controls: data normalization using the ^ΔΔ^C_T_ method of relative quantification; (3) miRNA reverse-transcription control (miRTC): assessment of reverse transcription performance; (4) positive PCR control (PPC): assessment of PCR performance.

We used a customized miRNA PCR chip, which is a high-flux detection tool that can be used to detect 84 miRNAs. The PCR array chip was used for screening tumor- related miRNAs; Table 1 and Figure 5 show the 84 miRNAs and their distribution in the chip; the 84 tumor-related miRNA primers were included on the 96-well plate. As explained in Figure 5, H3~H8 contained 5 housekeeping genes for microarray data standardization. H1 and H2 were genomic DNA references (GDC), in which fixed primer can specifically detect non-transcriptional genomic contamination in samples. H9 and H10 were repeated holes, all of which were reverse-transcription references (RTC), which were used to detect the efficiency of the RT reaction. H11 and H12 were positive PCR referenced (PPC), reflecting the efficiency of PCR reaction. The synthetic DNA sequences and the corresponding primer pairs were added to these holes. Two sets of repetitive control RTC and PPC can be used to detect the consistency within the chip and between the chips.

### 4.7. Statistical Analysis

All results obtained were expressed in mean ± SD. Statistical analysis was performed by *t*-test, using SPSS software version 21.0 (Beijing, China, 2015).

## 5. Conclusions

In summary, 100 mg/kg·day astaxanthin significantly inhibited PC-3 xenograft prostate tumor growth in nude mice by targeting multiple events and signaling pathways involved in apoptosis and proliferation. In addition, it might suppress tumor growth through inducing overexpression of miR-375 and miR-487b. These results provide a promising regimen to enhance the therapeutic effect in a dietary supplement manner.

## Figures and Tables

**Table 1:** List of 84 miRNAs for microarray analysis.

Tumor-Related miRNAs								
miR-1	miR-149	miR-200c	miR-32	miR-376c	miR-487b	miR-556-3p	miR-619	miR-744
miR-103-1as	miR-15b	miR-203	miR-320b	miR-377	miR-490-5p	miR-562	miR-625	miR-767-3p
miR-122	miR-154	miR-21	miR-320e	miR-378	miR-495	miR-571	miR-626	miR-885
mir124	miR-155	miR-210	miR-326	miR-381	miR-496	miR-574-3p	miR-627	miR-936
miR-126	miR-16	miR-218	miR-339-5p	miR-383	miR-512-3p	miR-575	miR-628-5p	
miR-135a	miR-182	miR-22	miR-345	miR-384	miR-517b	miR-591	miR-647	
miR-135b	miR-183	miR-221	miR-346	miR-432	miR-518c	miR-593	miR-654-3p	
miR-145	miR-185	miR-27b	miR-373	miR-449a	miR-548c-5p	miR-601	miR-661	
miR-146a	miR-193a-3p	miR-29a	miR-375	miR-485-3p	miR-551a	miR-608	miR-664	
miR-147b	miR-197	miR-299-5p	miR-376a	miR-486-5p	miR-554	miR-615-3p	miR-671-5p	
